# A Longitudinal Study of the Effect of Genistein on Bone in Two Different Murine Models of Diminished Estrogen-Producing Capacity

**DOI:** 10.4061/2010/145170

**Published:** 2009-10-18

**Authors:** Susan Reinwald, Loretta P. Mayer, Patricia B. Hoyer, Charles H. Turner, Stephen Barnes, Connie M. Weaver

**Affiliations:** ^1^Department of Foods & Nutrition, Purdue University, West Lafayette, IN 47907, USA; ^2^Department of Anatomy & Cell Biology, Indiana University School of Medicine, 635 Barnhill Drive, MS 5045B, Indianapolis, IN 46202-5120, USA; ^3^Department of Biological Sciences, Northern Arizona University, Flagstaff, AZ 86011, USA; ^4^Department of Physiology, University of Arizona, Tucson, AZ 85724, USA; ^5^Departments of Biomedical Engineering and Orthopaedic Surgery, Indiana University School of Medicine, IN 46202-3082, USA; ^6^Department of Pharmacology & Toxicology, University of Alabama, Birmingham, AL 35294, USA

## Abstract

This experiment was designed to assess the capacity of dietary genistein (GEN), to attenuate bone loss in ovariectomized (OVX) and ovary-intact VCD-treated mice. Pretreatment of mice with 4-vinylcyclohexene diepoxide (VCD) gradually and selectively destroys ovarian follicles whilst leaving ovarian androgen-producing cells largely intact. VCD induces a perimenopause-like condition prior to the onset of reproductive acyclicity. Sixteen-week-old C57BL/6J mice were randomized to five treatment groups: sham(SHM), OVX, SHM + VCD, OVX + GEN, and SHM + VCD + GEN. In vivo, blood samples were drawn for hormone and isoflavone analyses, estrous cycles were monitored, and X-ray imaging was performed to assess changes in bone parameters. Following sacrifice, ovaries were assessed histologically, bone microarchitecture was evaluated via microcomputed tomography, and bone mechanical properties were measured. Some effects of GEN were observed in OVX mice, but GEN effects were not able to be evaluated in VCD-treated mice due to the subtle diminution of bone during the 4 months of this experiment.

## 1. Introduction

Perimenopause is the transitional period in a woman's life characterized by the onset of irregular menstruation before permanent cessation of the menses or menopause. Estrogen (E2) insufficiency related to menopause has been shown to be a major pathological factor in the development and progression of osteoporosis, particularly in women that fall short of optimizing their genetic potential for peak bone mass in earlier adulthood. Although Premarin (conjugated estrogens) was the second most frequently prescribed medication in the United States in the year 2000 [1], its therapeutic use for the prevention or attenuation of bone loss in postmenopausal women is currently a controversial option given its potential for associated health risks [2, 3]. Based on outcomes of the Women's Health Initiative (WHI) study, the prescription of estrogen replacement therapy or estrogen in conjunction with progestin may not always be the best, safest, or preferred means by which to prevent pathologic bone loss during menopause [4]. General recommendations following the WHI stipulated that women should carefully weigh the benefits and risks of hormone replacement therapy and possibly consider nonhormonal options for the treatment of osteoporosis. Interest subsequently increased in various phytoestrogens, or natural plant-derived estrogen-like compounds, as a dietary therapeutic to facilitate the attenuation of bone loss [5]. Genistein (GEN) is one of a variety of isoflavone phytoestrogens derived from soybeans; it has been used in a number of food, beverage, and supplement formulations on the premise of delivering health benefits via its potential to act as a natural selective estrogen receptor modulator (SERM) [6] with partial estrogen receptor agonism that is less likely to perturb reproductive tissues.

To date, GEN research has yielded variable results and there is no consensus on the magnitude of its effect(s) on bone [7–10]. In human studies and animal experiments of estrogen deficiency, GEN has been shown to exert anabolic actions on bone [11–14] or attenuate bone loss via antiresorptive effects both as a purified compound [15–17] and as a component of soy isoflavone mixtures [18–20]. GEN has also exhibited anabolic effects on osteoblastic cells [21] and suppressive activities on osteoclasts [22, 23]. Overall, the therapeutic value of isoflavones to bone preservation has been more consistently demonstrated in ovariectomized rodents versus studies in postmenopausal women [24]. Some of the disparities in human versus rodent isoflavone studies may relate to the route of administration onset. 

In preclinical rodent experiments, bilateral ovariectomy is currently the most common method employed for bringing about a state of diminished E2 to simulate menopausal bone loss. However, ablation of the ovaries causes an unnaturally abrupt decline in circulating estrogens and ovarian-synthesized androgens. Inducing ovarian senescence via gradual follicle depletion may greatly improve the physiological relevance of hormone-linked bone loss experiments in animals by more accurately simulating the hormonal changes that take place in women as they transition to menopause [25]. Repeated chemical treatments with 4-vinylcyclohexene diepoxide (VCD) have been shown to induce progressive ovarian preantral follicle atresia in mice that gradually leads to a significantly diminished capacity for ovarian estrogen secretion [25–27]. Whereas OVX mice more closely simulate the hormonal profile of women that have undergone surgical menopause, VCD-treated mice remain ovary-intact with an established residual ovarian androgen-producing capacity [27, 28]. To date, one study by Wright et al. [25] has demonstrated significant bone loss in mice treated at a young age with VCD.

A summary of prospective studies on BMD changes during perimenopause indicates that annual percentage declines are often similar to those of women in the early stages of menopause when bone turnover is markedly elevated [29]. Other human data also provide evidence of significant bone loss during the perimenopausal period [30–33] which may be attributable to hypoestrogenic oligomenorrhea [34] (infrequent or light menstruation associated with low estrogen levels) experienced by many women and increasing gonadotropin levels [35]. Similar to hormone replacement therapy [36, 37], two human studies have suggested that dietary GEN may be more likely to demonstrate a favorable effect if administered earlier, rather than later on, in the menopausal period [11, 24]. Furthermore, it has been proposed that during perimenopause and early menopause estrogen receptor number and responsiveness may be more conducive to an isoflavone-mediated effect [38]. These considerations stimulated our interest in the VCD-treated mouse that transitions through a perimenopause-like period prior to reproductive acyclicity. The aim of this experiment was to assess the potential of GEN to attenuate bone loss in the traditional OVX mouse model and in the novel VCD-treated mouse that transitions to a menopause-like state.

## 2. Materials and Methods

### 2.1. Animals and Preparatory Procedures

Sixteen-week-old female C57BL/6J mice (total “*n*” of 100) with mean (±SD) body weights of 20.32 ±1.10 g were purchased from Jackson Laboratories (JAX, West Sacramento, CA). After purchase, all mice continued to be housed at JAX's animal facilities for a 3-week period so that preparatory treatments could be implemented prior to shipment.

In two of the treatment groups destined for sham surgeries (SHM), the chemical 4-vinylcyclohexene diepoxide (VCD) was administered by JAX animal technicians once daily for 15-d (starting day one) via intraperitoneal injections at a dose of 160 mg/kg/day (Sigma-Alrdrich; St. Louis, MO). A sesame oil vehicle was used as a placebo treatment for the remaining three groups. On day 18, three groups of mice underwent sham (SHM) surgeries and two groups were subjected to bilateral ovariectomy (OVX). In accordance with these initial treatments and the assigned GEN dietary regime for one SHM and one OVX group, rats were allocated to one of the following groups: (1) SHM, (2) OVX, (3) S + V (SHM + VCD), (4) O + G (OVX + GEN), and (5) SVG (SHM + VCD + GEN).

Upon arrival at our animal facility, mice were housed 5/cage, under conditions of controlled lighting (12-hour light:dark photoperiod), constant temperature (22 ± 2°C), and with free access to food and water. All mice were acclimated for 1-week prior to exposure to any type of procedures/measurements.

### 2.2. Diets

From day one of the experiment, all five groups of mice (16-wk-old) were fed a casein-based diet (5K96; Testdiet, Richmond, IN) formulated to consistently measure <1 ppm total isoflavones (aglycone equivalents of genistein, daidzein, and glycitein). Following OVX/SHM surgeries, at which time the mice had reached 18-wk-old, two groups of mice (i.e., O + G and SVG) were supplemented with the aglycone form of the isoflavone GEN. The GEN was delivered to mice via the oral route as a 0.04% (w/w) constituent of the pelleted 5K96 diet supplying ~55 mg GEN/kg body weight/day. This represents the average level of the dosage range reported to stimulate bioactivity in rodents (i.e., 10 to 100 mg/kg bwt/day) [39]. The GEN used to enrich the diet was in crystalline powder form and >99% pure (Product # G-6055, LC Laboratories, Woburn, MA).

### 2.3. Experimental Design

**Figure 1:**
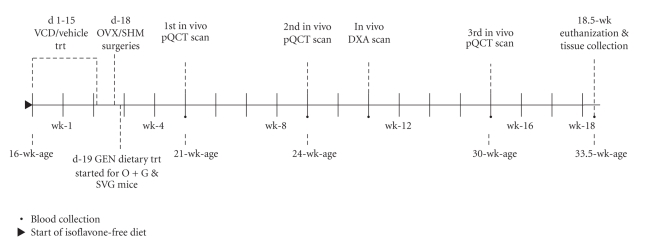
Experimental timeline.

This study was longitudinal in design; in vivo bone imaging and blood sample collections were performed at intervals depicted in the experimental time line (Figure 1). The body weights of mice were recorded weekly and prior to all experimental procedures from the start of the experiment until the time of euthanization (18.5-wk later). Sacrifice was via exsanguination followed by cervical dislocation under anesthesia when the mice were 33.5-wk-old. All experimental procedures complied with protocols were approved by Indiana University School of Medicine and Purdue University Care and Use Committee guidelines.

### 2.4. Reproductive Cyclicity

Estrous cycle stage was determined by vaginal cytology in SHM (cycling control), S + V, and SVG treatment groups throughout the experiment to verify the disruption and eventual cessation of normal estrous cycling resulting from VCD treatment. Acyclicity was defined as 10 consecutive days of diestrus. A vaginal lavage technique was used for cytology evaluations.

### 2.5. Bone Imaging

#### 2.5.1. In Vivo DXA

A Lunar PIXImusII densitometer (GE Medical Systems) was utilized to obtain data from projectional images of the right hindlimb of mice to assess areal BMD (mg/cm^2^), BMC (mg), and bone area (cm^2^) 8-wk after ovariectomy. BMC was normalized by body weight (BWT) to adjust for differences in body size [40]. Mice were immobilized for the scanning procedure by gas inhalation (1.5–2.0% isoflurane in 1.5 L/min O_2_) and placed in prone position on the scanning stage. The entire right hindlimb region, up to but excluding the pelvic girdle, was selected for analysis rather than individual bones (i.e., femora, tibiae). The coefficients of variation (CV%) for DXA analyses (BMD, 0.73%; BMC, 1.16%; area, 0.97%) were based on 10 consecutive in vivo hindlimb scans with complete repositioning of the animal between scans.

#### 2.5.2. In Vivo pQCT

**Figure 2:**
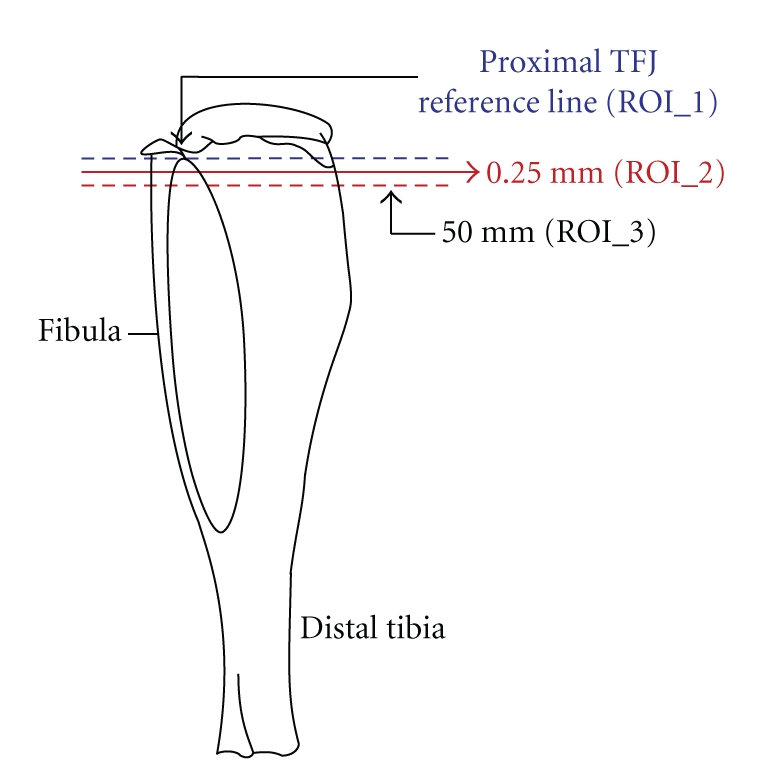
pQCT regions of interest in the proximal tibiae of mice. Distances of scanned slices from the proximal tibiofibular junction.

The proximal metaphyses of the left tibiae of mice were measured longitudinally by repeated in vivo peripheral quantitative computed tomography (pQCT) scans at 5-, 9-, and 15-wk of the experiment. Each mouse was anesthetized (i.e., 3%–4% isoflurane in 1.5 L/min O_2_) for induction to effect before being placed in a supine position on a heated pad with its nose exposed to a tube supplying isoflurane gas (i.e., 1.5%–2.0% in 1.5 L/min O_2_) to maintain light anesthesia. The left hindlimb was extended in the caudal direction and centered and secured with adhesive tape onto a 1 cm wide support that protruded into the pQCT gantry. The right forelimb was extended cephalically and taped in place to counterbalance, align, and further secure the animal's body during scanning. Based on an initial cross-sectional scout-scan, a first landmark reference slice (ROI_1) was placed under the growth plate in alignment with the most distal aspect of the proximal tibiofibular junction (TFJ) (Figure 2). Images for analyses were obtained from a second and third slice placed 0.25 mm (ROI_2) and 0.50 mm (ROI_3) distal to the first slice, respectively. Correct targeting of the ROIs was confirmed when the ROI_1 image displayed contiguity between the proximal tibia and fibula, and when the RIO_2 image from the same scan series exhibited the separation of these two bones from each other. 

Single slice cross-sections of bone 0.26 mm thick were imaged with a XCT Research SA + pQCT (Norland-Stratec Medizintechnik GmbH, Birkenfel, Germany) at an isotropic voxel resolution of 70 *μ*m. The pQCT was programmed to use cortmode 1 to separate cortical voxels from trabecular voxels, contmode 1 to detect the outer bone contour, and peel mode 2 to define the trabecular compartment. An attenuation threshold of 900 mg/cm^3^ was selected to distinguish cortical from trabecular bone [41], whereas soft tissue was differentiated from bone at a threshold 300 mg/cm^3^. Analyses of pQCT images were performed using the manufacturer's integrated XCT software package (version 5.50) that records parameters in a loop project.

A CV% was calculated (i.e., 100 × [standard deviation/mean]) based on the average values of 20 separate pQCT scans (with repositioning between each scan) acquired at each ROI. Five in vivo scans per animal in four mice were performed for this purpose. The CVs at ROI_2 for total, cortical, and trabecular BMD were 1.6%, 1.6%, and 2.1% respectively. The CV for cortical thickness at ROI_2 was much higher at 27.2%.

#### 2.5.3. Ex Vivo *μ*CT

A high-resolution desktop microtomographic imaging system with a microfocus X-ray tube as a source (*μ*CT40; Scanco Medical, Basserdorf, Switzerland) was used to image contiguous transverse slices of trabecular bone at the distal femoral metaphysis and the fifth lumbar vertebral body (L5) as well as cortical bone at the femoral midshaft. Measurements at each location on the femur and vertebral body were performed on six randomly selected samples per treatment group at a nominal isotropic voxel resolution of 6 *μ*m and 12 *μ*m, respectively. Images were segmented using a constrained 3D Gaussian filter (sigma = 0.8 and support = 1 pixel). Site-specific thresholds that were 30% and 22.4% of the maximal gray-scale value were selected to adjust for variations in absorption coefficients between cortical and trabecular bone, respectively [42]. A 50-slice region spanning 300 *μ*m in the midshaft of the femoral diaphysis was scanned. Trabecular measurements in the distal femoral metaphysis were confined to 250 slices (a region of 1500 *μ*m in length) in the secondary spongiosa located 408 *μ*m proximal to the physeal-metaphyseal junction landmark. Microtomographic images were collected over a 2220 *μ*m long mid-vertebral region between the cranial and caudal endplates of the L5 vertebral body. Trabeculae of the left distal femoral metaphysis and fifth lumbar vertebral body (L5) of mice were evaluated for bone volume fraction (BV/TV, %) and direct 3D indices including trabecular thickness (Tb.Th, *μ*m), trabecular separation (Tb.Sp, *μ*m), and trabecular number (Tb.N, mm^−1^). *μ*CT cortical bone parameters assessed included volume (BV, mm^3^), area (BA, mm^2^), and cortical thickness (Ct.Th, mm). 

The *μ*CT CV% (based on 10 repeat scans of one sample with complete repositioning between scans) acquired at each site of interest revealed a precision of <3% and <1%, respectively, at the distal metaphysis and midshaft of the femur for all parameters reported. The CV% was <2% for all vertebral *μ*CT parameters, with the exception of 4.2% for BV/TV.

### 2.6. Three-Point Biomechanical Testing

Mechanical testing was performed on a miniature materials-testing machine (Vitrodyne V1000: Liveco, Inc., Burlington, VT, USA) with a force resolution of 0.05 N. All bones for mechanical testing were stored at −20°C. Prior to testing, samples were defleshed and rehydrated overnight in 0.9% saline at room temperature [43]. Supports on which the bones rested during testing spanned 8.5 mm and 11.2 mm for the right femora and left tibiae, respectively. A small compressive stabilizing preload of <0.1 N was applied to specimens prior to the start of the test. Bones were loaded centrally in the most stable orientation, (i.e., anterior to posterior for the femur, and posterior to anterior for the tibia) at the midshaft of the diaphyses using a crosshead speed of 0.2 mm/sec. The yield point was defined using a 0.015 mm offset parallel to the linear elastic region of the curve. Force-displacement data, collected by the software every 0.02 sec during each test, was imported into an Excel worksheet formatted to generate the structural parameters and mechanical properties of the bones. These included ultimate force (N), failure (breaking) force (N), yield force (N), stiffness (N/mm), and energy to ultimate force (mJ), energy to failure (mJ). Total bone length (mm), measured using digital calipers (Mitutoyo, 0.01 mm resolution), was also included with bone mechanical data.

### 2.7. Blood Collection

Small quantities of blood were collected periodically (≤200 *μ*L at >14-day intervals [44]) in Microtainer Plasma Separator Tubes (Beckton-Dickson, NJ, USA) during the experiment at time points coinciding with in vivo x-ray scan measurements. A submandibular vein puncture technique was employed for all survival blood collections. Light anesthesia was induced via gas inhalation (Isoflo, Abbott Laboratories, North Chicago, IL) after which the mice were momentarily immobilized and scruffed and the underlying vasculature was pierced with a lancet (GoldenRod, Medipoint Inc. Mineola, NY, USA) to draw blood droplets. To permit collection of the maximum volume of blood available at the end of the study, a terminal retro-orbital exsanguination procedure was performed while the animals were under deep sedation.

### 2.8. Hormone Analysis

Plasma levels of androstenedione (ANDRO) and 17*β* estradiol (E2) were determined via duplicate assays using radioimmunoassay (RIA) kits (Diagnostic Products Inc., Los Angeles, CA). Follicle stimulating hormone (FSH) plasma levels were determined using a National Hormone and Pituitary Distribution Program RIA kit. Results of all RIAs were calculated by four-parameter logistic analysis using the AssayZap software program (Biosoft, Ferguson, MO). Respective sensitivity and intraassay coefficients of variation were 17*β*-estradiol, 1.25 pg/mL, and 2.46%; androstenedione: 30 pg/mL and 7.25%; FSH: 100 pg/mL and 1.71%.

### 2.9. Isoflavone Analysis

Plasma isoflavone concentrations were determined by reverse phase LC-electrospray ionization mass spectrometry on a triple quadrupole mass spectrometer (ABI-Sciex 4000 Qtrap LC/MS/MS system, Applied Biosystems, San Jose, CA) using a modification (i.e., liquid-liquid extraction) of the method described previously by Coward et al. [45]. Due to the very small aliquots of plasma available from each mouse, two pooled samples from each cage were analyzed and results were averaged.

### 2.10. Statistics

The SAS statistical program (version 9.1, SAS Institute, Cary, NC) was used to analyze all data. Homogeneity of variance (Levene's test) and normality (Shapiro-Wilk's W test) were routinely assessed to ensure that the assumptions of ANOVA tests were met. One-way ANOVA was used for parametric data, and when differences were statistically significant (*P* < .05), pairwise comparisons among means were performed using the post hoc Tukey-Kramer procedure. Nonparametric data were analyzed using the Kruskal-Wallis test (the nonparametric analog of a one-way-ANOVA). Statistically different nonparametric tests (*P* < .05) were compared using Dunn's post hoc test. Data are reported as group means ± SEM unless stated otherwise.

## 3. Results

### 3.1. Overview of the Animals

Four mice died before being shipped from JAX laboratories and two mice died during the study. Records documenting the general outward appearance, health, and behavior of the mice were maintained throughout the experiment. At necropsy it was determined that some mice had gross gonadal abnormalities (see ovary results); however, affected animals were eliminated from all but the food consumption analyses due to the nature of the group-housed data. As a consequence, group data were limited to SHM (*n* = 11), OVX (*n* = 15), S + V (*n* = 12), O + G (*n* = 18), and SVG (*n* = 12) mice.

### 3.2. Body Weights

**Figure 3:**
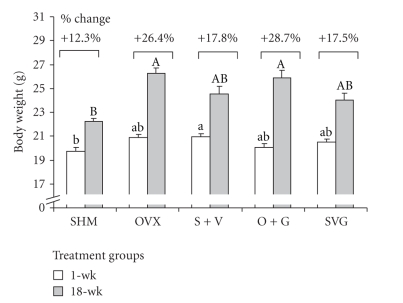
Mouse body weights at the beginning and end of the study. Bars represent mean ± SEM values. Different lower- and upper-case letters signify differences between groups at 1-wk (*P* = .0212) and 18-wk (*P* < .0001), respectively. Percentage values correspond to the mean changes in body weight over the duration of the experiment for each of the treatment groups.

Mean body weights were significantly different among groups at the beginning and end of the study (Figure 3 **)**. Initially S + V mice weighed significantly more than the SHM rats and no other groups were different from the SHM and S + V treatment groups. At the end of the study ovariectomized mice (OVX and O + G) had gained the highest percentage of weight and were significantly heavier than the SHM group, but not different from VCD-treated mice.

### 3.3. Food Consumption

Ovariectomy increased the amount of food consumed in the OVX group compared to the SHM and S + V mice (*P* < .05; data not shown). No food consumption differences existed between other treatment groups. Daily genistein consumption, normalized for body weight, was comparable between O + G and SVG mice.

### 3.4. Isoflavone Analysis

**Figure 4:**
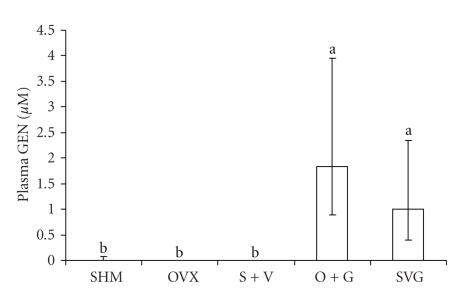
A comparison of plasma GEN concentrations as a result of dietary treatments in mice. Data represent the group median and error bars indicate the interquartile range for samples collected after 18-wk GEN or no-GEN in the base diet. The Kruskal-Wallis test for nonparametric data revealed a significant difference between groups (*P* < .0001), and values with a different letter are statistically different based on Dunn's post hoc multiple comparison procedure.

Blood collected from mice during the terminal blood draw at 18-wk was analyzed for plasma concentrations of numerous isoflavones including genistein (GEN), daidzein, dihydrodaidzein, *O*-desmethylangolensin (O-DMA), equol, glycitein, formononetin, coumestrol, biochanin A, and 6-OH O-DMA. With the exception of the highly significant differences in the plasma GEN concentrations among groups (*P* < .0001) that were fed versus not fed a GEN-enriched diet (Figure 4), no differences were detected among treatment groups for the other isoflavones analyzed (data not shown). Plasma GEN levels in O + G and SVG treatment groups were not statistically different from each other.

### 3.5. Reproductive Cyclicity

The S + V and SVG mice that were administered VCD for 15 consecutive days were observed to cease reproductive cycling at a mean(±SEM) of 80 ± 9 and 86 ± 8 days after the initial VCD injection (*P* > .05; data not shown). By comparison, the SHM mice included in all analyses demonstrated relatively regular estrous cycles that ranged from 4 to 5 days in duration.

### 3.6. Uterine Weights

Both ovariectomy and VCD treatment resulted in uterine atrophy relative to the SHM mice (*P* < .0001). Ovariectomy caused the greatest mean decrease in uterine weight (>75% versus SHM) while VCD treatment reduced mean uterine weight by at least 50% versus SHM. Groups treated with GEN (O + G and SVG) did not exhibit increased uterine weight/body weights compared to their non-GEN-treated counterparts, indicating that GEN did not exert a uterotrophic effect in this study (data not shown).

### 3.7. Ovaries

At necropsy, ovaries of intact mice were removed from the surrounding fat pad for further analyses. Some pathologic abnormalities in a number of ovaries were observed at this point; a number of mice developed substantial ovarian fluid-filled bursal sac expansions (ranging in diameter from 0.5- to 1.0 cm), some unilaterally, some bilaterally, that were associated with severely atrophied ovarian tissue at the affected site(s). Mice belonging to the SHM, S + V, and SVG groups were affected (*n* = 8, *n* = 7 and *n* = 7, resp.) and these animals were eliminated from the analyses. Also, two OVX mice identified as having an ovary inadvertently left in place and two O + G mice with large fluid filled sacs at the end of the fallopian tubes but with no ovarian tissue present were excluded from the results.

**Figure 5:**
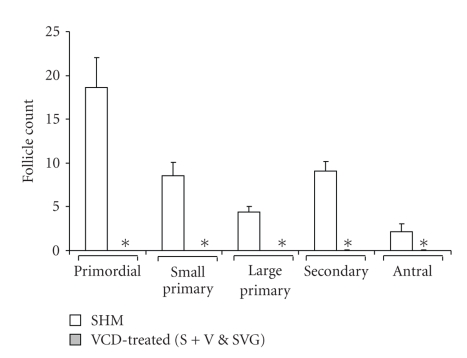
Effect of VCD-treatments versus controls on follicle type counts in mouse ovaries. Columns represent the mean total number of follicles counted in every 20th section (5- *μ*m) of each ovary and error bars indicate the SEM for *n* = 11 SHM and *n* = 23 VCD-treated mice (S + V and SVG). Data from S + V and SVG groups were determined not to be significantly different from one another, based on a *t* test, and were pooled for comparison against SHM controls. * indicates significance (*P* < .0001) for SHM-versus VCD-treated mice for each follicle type using the Mann-Whitney test.

Ovaries from the remaining healthy animals underwent sectioning for histological follicle counts (Figure 5). Since there was no significant difference between the VCD-treated S + V and SVG mice (based on an F-test that met ANOVA assumptions), they were grouped together for comparison against the SHM controls using the nonparametric Wilcoxon-Mann-Whitney Test which revealed a significant difference between control and VCD-treated mice (*P* < .0001) for all types of follicle counts performed (i.e., primordial, small primary, large primary, secondary, and antral). Ovaries were also examined for corpora lutea; the ovaries of cycling mice revealed the presence of corpora lutea (100%), whereas they were absent from all VCD-treated mice. SHM mice were generally characterized by the presence of antral follicles, whereas only one of the total number of VCD-treated mice exhibited the presence of an antral follicle (S + V), which was observed to be atretic (i.e., degenerated) and therefore included in all analyses.

### 3.8. Hormone Analyses

**Figure 6:**
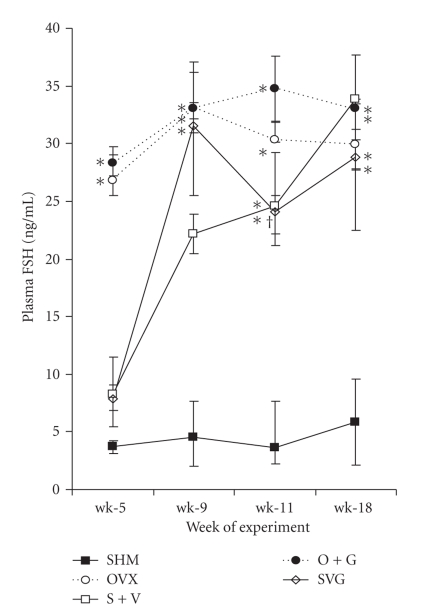
Comparisons of plasma FSH among groups of mice. One-way ANOVAs performed at each time period were significant (*P* < .0001). Values represent means ± SEM. Different symbols signify differences among groups based on the Tukey-Kramer post hoc procedure as follows: *  SHM< all other groups; ^†^  VCD-treated mice < OVX groups; ^§^  S + V < OVX groups; ^‡^  SVG < O + G.

Plasma concentrations of FSH remained consistently low in the SHM control mice throughout the experiment (Figure 6). FSH concentrations in ovariectomized mice (OVX and O + G) spiked markedly by wk-5 and remained elevated throughout the remaining weeks of the experiment. VCD-treated mice displayed elevated concentrations of FSH that were significantly higher than sham controls at 5-wk, yet significantly lower than ovariectomized mice. During wk-9 to wk-11 FSH gradually began to increase as the animals transitioned to a menopause-like state and by wk-18 the VCD-treated mice had circulating FSH concentrations comparable to the OVX mice. 

**Figure 7:**
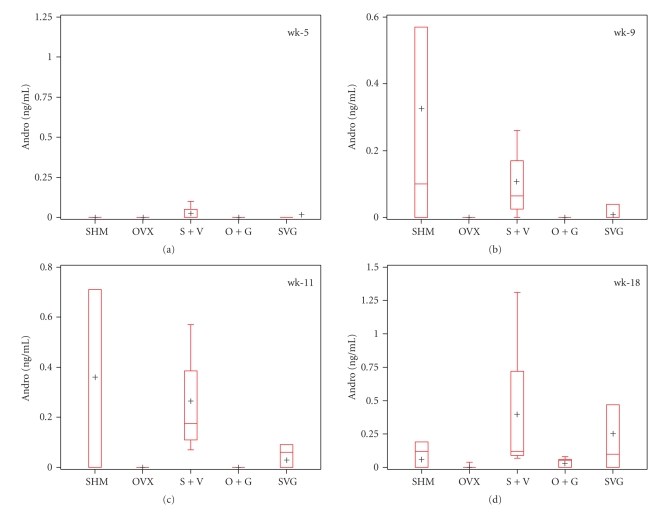
Treatment effects on plasma androgen (ANDRO) concentrations of mice. The panel of boxplots illustrates the group medians (line within the box), means (+), interquartile range (length of the box), and the minimum and maximum values (extended whiskers) at each time point assessed. Data represent *n* = 3–6 samples/group. The Kruskal-Wallis test revealed the following *P*-values: wk-5, *P* = .3732; wk-9, *P* = .0387; wk-11, *P* = .0142; wk-18, *P* = .0593.

Circulating concentrations of 17*β*-estradiol averaged as high as 6.35 pg/mL and 4.86 pg/mL in S + V and SVG mice (*n* = 4/group), respectively, at wk-11, after which levels plummeted to nondetectable levels by wk-18. The 17*β*-estradiol data are not presented as it is somewhat difficult to interpret due to differences in estrous cycle stage among animals as they transitioned to reproductive senescence. OVX and O + G mice (*n* = 6-7/group) synthesized comparatively low average levels of E2 at the time points we measured; however, by wk-11 in O + G and by wk-18 in OVX and mice, E2 was below the detection limit and only above the detection limit in sham controls. Plasma ANDRO concentrations from wk = 9–18 were detectable in most SHM-and VCD-treated mice and relatively undetectable in ovariectomized mice (Figure 7). 

### 3.9. DXA

Ovariectomy, both with and without GEN treatment, decreased BMD and BMC normalized for body size compared to SHM 8-wk postovariectomy (Table 1). There was no difference between BMD and BMC/BWT for VCD-treated versus SHM mice. Bone area was not different among treatments.

### 3.10. pQCT

**Figure 8:**
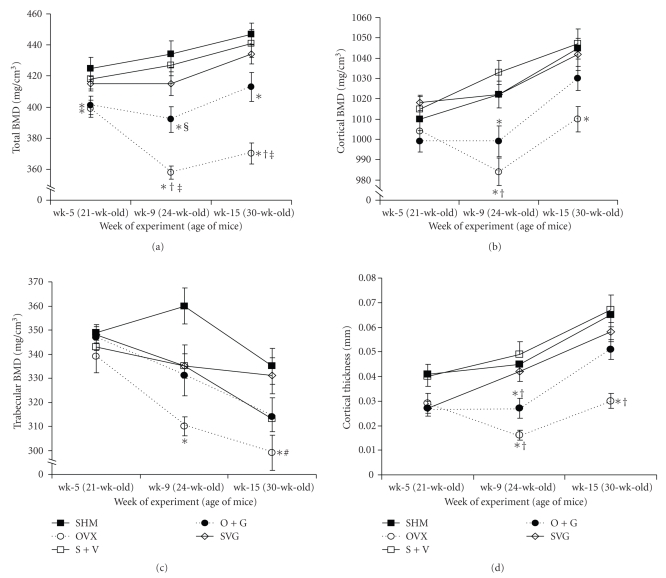
Treatment effects on the bone mineral density and cortical thickness of the proximal tibial metaphysis as assessed longitudinally in vivo via pQCT. Data represent the mean ± SEM values imaged 0.25 mm distal to the tibiofibular junction. Symbols indicate significantly different means (*P* < .005) as assessed by a one-way ANOVA and subsequent post hoc Tukey tests as follows: * group versus SHM; ^†^  group versus VCD-treated mice (S + V and SVG); ^§^  group versus SVG; ^‡^  group vs. O + G; ^#^ group versus SVG.

Longitudinal pQCT scanning data from the two ROIs below the reference line at the tibiofibular junction (TFJ) revealed similar differences between the two ROI sites. Herein we present data from 0.25 mm below the TFJ (Figure 8). GEN appeared to be associated with an increase in total BMD, cortical thickness, and cortical density by the time of the last pQCT scan in O + G versus OVX mice. The VCD-treated mice retained a similar cortical thickness and BMDs similar to SHM mice up to 30-wk-age or 15-wk after the initiation of VCD dosing. A coefficient of variation (CV%) was calculated (i.e.,100 × [standard deviation/mean]) based on the average values of 20 separate pQCT scans (with repositioning between each scan) acquired at each ROI. Five in vivo scans per animal in four mice were performed for this purpose. The CVs at ROI_2 for total, cortical and trabecular BMD were 1.6%, 1.6%, and 2.1%, respectively. The CV for cortical thickness at ROI_2 was much higher at 27.2%; however, all significant differences among groups for cortical thickness were almost twice the CV% for this parameter.

### 3.11. *μ*CT

Trabecular bone architecture in the distal femur was similar among all groups of mice regardless of SHM/OVX status (16-wks duration) and/or VCD treatment (18-wk after the start of dosing) and/or dietary GEN supplementation (for 18-wks). In contrast, trabecular bone in the fifth lumbar (L5) vertebral body exhibited a number of differences among treatments (Table 2). L5 bone volume fraction (BV/TV) in OVX and O + G groups was decreased compared to SHM mice. The S + V mice retained a comparable vertebral BV/TV versus SHM controls; however, this parameter was compromised in SVG mice versus SHM. Fifth lumbar Tb.Th was equivalent among all groups with the exception of the OVX mice in which trabecular thinning was detected. The OVX mice that received GEN (O + G) appeared to have been protected against this occurrence. Apart from differences in BV/TV, the VCD-treated mice were similar to SHM mice with respect to vertebral trabeculae morphology. At the midshaft of the femur, the OVX mice displayed a significantly diminished cortical envelope compared to all other groups, with the O + G group of mice appearing to have been protected against cortical thinning by the addition of dietary GEN. Cortical bone area and volume at the midshaft was also significantly decreased in OVX mice compared to SHM, whereas other treatment groups were not. 

### 3.12. Mechanical Testing

Three-point bending tests of the tibiae did not resolve any differences in whole bone strength parameters pertaining to ovariectomy, VCD treatment, or dietary GEN exposure under our test conditions (Table 3). Tibia length was increased in OVX mice relative to SHM and SVG mice. VCD-treated mice exhibited similar bone lengths to SHM animals. Femur mechanical testing revealed that the OVX mice had less cortical bone strength as evidenced by the significant reductions in yield force, ultimate force, and failure force parameters compared to SHM controls. Dietary GEN was associated with an increase in the ultimate load withstood by the femoral midshaft in O + G versus OVX mice. The structural strength of the femurs of VCD-treated mice remained similar to SHM mice 18-wks after the initiation of VCD dosing, and it was not possible to assess any effects of GEN in these animals at that endpoint.

## 4. Discussion

This study examined two different murine models of diminished estrogen-producing capacity that reach a menopausal-like state. The first is the traditional OVX mouse that is a model of surgically-induced menopause. The second more recently developed VCD-treated mouse is a model of chemically-induced menopause. The goal of this study was to assess the capacity of GEN supplementation to attenuate bone loss in each of these models. Whereas the amount of bone loss in mice attributable to ovariectomy-induced menopause was significant in relation to the SHM controls, the bones of VCD-treated mice did not appreciably change in relation to SHM controls over the duration of this experiment. These results suggest that, in the absence of ovarian estrogen, ovarian testosterone in VCD-treated mice may attenuate bone loss and delay detrimental changes in the skeleton. GEN was associated with some positive effects on the bones of O + G mice compared to OVX mice including a higher trabecular BMD (proximal tibia) as well as improvements in trabecular (vertebral) and cortical (femoral) architecture and a structural strength parameter (femur) comparable to SHM levels. The downside to assessing the effects of GEN in the OVX model is that it simulates an unnatural and sudden transition to menopause. Less than 13% of women undergo surgical oophorectomy which results in immediate sex hormone depletion [46]. The relative lack of bone loss in VCD-treated mice versus SHM controls during the chemically-induced perimenopausal-menopausal period raised some interesting questions in relation to estrogen-deficient animal models for bone research. However, during the time of this experiment it was not possible to establish any effect of GEN on VCD-treated mice. 

Unlike most other bone studies in mice in which the effects of GEN have been evaluated following subcutaneous delivery via osmotic pumps or injection regimens [15–17, 47, 48], this study assessed the effects of daily GEN consumption on bone. GEN administered at 0.4 g/kg of food to treated mice resulted in consumption levels of 55 mg GEN/kg body weight, or on average 1.2 mg GEN/day. This dose is in keeping with other rodent studies in which 42 mg [49] and 50 mg dietary GEN/kg bodyweight/day [50, 51] was evaluated for bone-sparing effects following ovariectomy. Anderson et al. [52] classified dietary GEN supplementation in rats as low (0.5 mg/day), intermediate (1.6 mg/day), and high (5.0 mg/day) when assessing changes in bone tissue. To put these supplementary GEN doses into some context with respect to human intakes, traditional East-Asiatic diets that are soy-rich supply isoflavones ranging from 1 mg/kg bodyweight/day [39] to 25-100 mg/day [53], whereas the typical West European and North American diet only supplies a few milligrams of isoflavones per day. Human observational studies suggest that isoflavone effects on BMD are evident in women consuming >40–60 mg/day [54] whereas human intervention trials demonstrate bone-conserving effects in women supplemented with 54 mg GEN/day or up to 80–99 mg isoflavones/day [19, 55, 56].

While VCD-treated mouse bones remained similar to those of SHM controls for the duration of this study, VCD-treated mice did appear to better simulate the human physiological transition to menopause. S + V and SVG mice underwent a perimenopausal-like period characterized by significant estrous cycle perturbations compared to SHM controls based on the vaginal cytology analyses. The VCD-treated mice also exhibited a gradual or time-dependent increase in the secretion of the gonadotropin FSH compared to OVX mice. This occurs as a result of diminishing levels of ovarian-synthesized estrogen and inhibin-A episodically disrupting the negative feedback loop governing the hypothalamic-pituitary-gonadal axis such that the pituitary persists in secreting FSH [57]. The VCD-treated mice mimicked the temporal rise in FSH observed in women that naturally transition to menopause. Evidence exists to suggest that both ovulation disturbances and elevated gonadotropin levels during perimenopause are associated with accelerated bone loss in women [29, 58]. With the exception of the decrease in vertebral trabecular BV/TV in SVG relative to SHM mice, significant perimenopausal bone loss was not observed in the VCD-treated mice in this study. 

Wright et al. [25] have compared the skeletal effects of VCD-treated C57Bl/6Hsd mice that took between 38 and 64 days to become acyclic, to ovariectomized and ovary-intact control mice. Their data indicate that while spinal BMD (mg/cm^2^) in VCD-treated mice took 2.9 months after the event of ovarian follicle depletion to decrease relative to controls, age-matched OVX mice exhibited a decreased spinal BMD versus controls as soon as 1 months after ovariectomy. By 3.4-months postovarian follicle depletion or OVX, the decline in spinal BMD was similar for VCD-treated and OVX mice and both models exhibited BMDs that were comparably lower than control mice. These results are difficult to compare to our findings considering that the mice in the Wright et al. study were dosed with VCD as early as 28-day-old, whereas the mice in our study were dosed at an age when they are considered to be more skeletally mature. Given that VCD treatment of sexually immature mice makes it problematic to determine what changes in bone density and structure are attributable to perturbations in growth and development as opposed to bone loss, older mice may represent a more age-appropriate model for osteoporosis research. Interestingly, the longitudinal pQCT data collected for our older mice demonstrated that while cortical BMD of the tibiae continued to increase in mice up to at least 30-wk-age, trabecular BMD at the same location declined over the duration of the experiment. 

Ex vivo *μ*CT analysis performed by Wright et al. in bones of 5.3-months postmenopausal (i.e., 8.5-months-old VCD-treated) and intact control mice revealed no significant differences in trabecular microarchitecture at the distal femur and in cortical bone parameters at the femoral midshaft. The fact that our *μ*CT data showed correspondingly low BV/TV values in 8.5-months-old mice (on average 1-months after ovarian follicle depletion) and considering no difference between control and VCD-treated mice at the distal femur was evident collectively suggests that this site may undergo extensive trabecular bone loss due to age in this mouse strain making differences more difficult to detect. The *μ*CT data are also similar between studies in relation to VCD-treated and ovary-intact controls at the femoral midshaft, but not at the 5th lumbar vertebra. Whereas diminished trabecular morphology was associated with early VCD-treatment in the Wright study, it was not different in the mature VCD-treated versus SHM mice in the present study 1-month after ovarian follicle depletion. The shorter duration of ovarian follicle depletion in our VCD-treated mice may explain this difference. Further investigation of skeletally mature VCD-treated rodents beyond 1-month of ovarian follicle depletion is warranted to evaluate the practical utility of this model for osteoporosis research. 

Androgens have been determined to have a positive effect on BMD in postmenopausal women [59]. Research published by Rivera et al. [28] confirms the continued androgen-producing capacity of follicle-depleted ovarian tissue following VCD treatment of mice. A major shortcoming associated with the OVX rodent model that is frequently overlooked when assessing the efficacy of therapeutics to attenuate bone loss relates to ovary ablation abolishing the potential for ovarian androgen synthesis during the menopause-like state. Activity of the enzyme 17*α*-hydroxylase/17,20-lyase cytochrome P450 (P450c17) is required for the production of androgens in all animals [60]. While humans express P450c17 in the adrenals and gonads, rodents express p450c17 predominantly in the gonads and it is absent from the adrenals [61, 62] beyond the embryonic stage of life [63]. The state of estrogen [64] and androgen depletion [25] attributable to ovariectomy of rodents therefore generates a relatively more severely compromised sex steroid status than is typical in either natural menopause or oophorectomy for women. The concentrations of the androgen androstenodione were wide ranging in the blood samples collected from SHM, S + V, and SVG mice during wk-9 to wk-18, yet clearly most prominent in the ovary-intact mice. The presence of androgens in the VCD-treated mice is likely implicated in their similarity of bone parameters to control rats, as opposed to the differences in bone parameters between OVX and controls.

In contrast to many women, the lack of an appreciable amount of bone loss during the perimenopausal transition to menopause and during early menopause in the VCD-treated mice could be attributable to a number of factors. These may include the mouse strain, sample sizes, the VCD dose/regimen for the age of the mice, and/or the superlative nutritional history of standard laboratory rodents. Many rodent diets, especially natural ingredient diets, generally contain ≥1% dietary calcium which is at least twice the estimated recommended Ca intake for rodent maintenance [65, 66]. Traditionally, this situation is unlike one experienced by the majority of women that habitually struggle to meet adequate Ca intake requirements throughout their adult life [67, 68]. Manipulation of factors such as these could further influence the results of this experiment.

According to the existing body of research, a carefully regulated VCD dosing regimen for rodents specifically targets oocyte-containing small preantral ovarian follicles without toxic effects or abnormalities manifesting in other cells, tissues, or organs [25, 27, 69, 70]. Fluid-filled ovarian cysts are not uncommon in mice, especially as they age and total ovulation number increases [71]. The ovary abnormalities observed in some of the mice in this study were not specific to VCD treatment and may not have even been pathological in nature. Sentinel mice housed in the same room of the facility were assessed for potential pathogens and passed a stringent necropsy inspection report. However, as a precautionary measure, bone and other data of affected animals were summarily omitted from all but the food intake analyses due to group housing.

## 5. Conclusion

Our study may be the first to demonstrate that dietary GEN, as opposed to injected GEN, provides some modest benefits to the bones of skeletally mature ovariectomized mice. However, considering that the OVX mouse is an instantaneous model of menopause and negates the postmenopausal contributions made to the skeleton by ovarian androgens, this makes it difficult to translate what this may mean for the majority of women that remain ovary-intact and gradually transition to menopause. The VCD-treated mouse better simulates the hormonal milieu and reproductive cycling changes commensurate with perimenopause and menopause in women. Yet, based on this investigation, appreciable changes in bone in response to a VCD-augmented transition to menopause in mature animals did not occur up to one month after ovarian follicle depletion. Although this did not provide an opportunity in which to assess the skeletal effects of dietary GEN in the VCD-treated mice, we acknowledge that many potential manipulatable aspects of this rodent model remain unexplored. With these possibilities in mind, further investigation is required to determine how applicable the VCD model may be for osteoporosis research. As a rodent model to best simulate human menopause, it may be far superior provided that researchers can take on the additional cost and time required for VCD-treated mice to experience significant bone loss.

## Figures and Tables

**Table 1:** DXA parameters of the hindlimb assessed in vivo.

Parameters, (units)	SHM	OVX	S + V	O + G	SVG
BMD, (mg/cm^2^)	49.9 ± 0.4	46.7 ± 0.4*	48.1 ± 0.6	46.9 ± 0.9*	48.8 ±0.4
BMC/BWT	2.74 ± 0.03	2.35 ± 0.04**	2.69 ± 0.03	2.45 ± 0.05**	2.65 ± 0.04
Area, (cm^2^)	1.160 ± 0.001	1.211 ± 0.011	1.195 ± 0.001	1.206 ± 0.014	1.193 ± 0.019

Data represent the mean ± SEM. *P < 0.001 versus SHM; **P < .0001 versus SHM-and VCD-treated groups.

**Table 2:** Ex vivo microcomputed tomography measures of bone microarchitectural changes in treated mice.

*μ*CT parameters (units)	Treatments (*n* = 6/group)							
	SHM	OVX	S + V	O + G	SVG		Pooled SEM	*P*-value
L5 Vertebral body: trabecular								
BV/TV, (%)	18.4^a^	12.4^c^	15.1^ab^	14.5^bc^	15.7^b^		0.6	<0.0001
BV, (mm^3^)	0.472^a^	0.354^b^	0.394^ab^	0.396^ab^	0.426^ab^		0.019	<0.005
Tb.N, (mm^−1^)	3.32^a^	2.77^b^	2.94^ab^	2.76^b^	2.97^ab^		0.116	<0.05
Tb.Th, (*μ*m)	55^a^	47^b^	55^a^	56^a^	55^a^		1	<0.0001
Tb.Sp, (*μ*m)	305	367	342	367	341		16	NS
Distal femur: trabecular								
BV/TV, (%)	2.62	2.05	2.39	1.90	2.00		0.08	NS
BV, (mm^3^)	0.066	0.057	0.062	0.46	0.53		0.01	NS
Tb.N, (mm^−1^)	2.21	1.94	2.01	1.91	2.07		0.20	NS
Tb.Th, (*μ*m)	41	42	44	43	40		2	NS
Tb.Sp, (*μ*m)	446	522	500	524	483		22	NS
Midshaft femur: cortical								
Area (mm^2^)	0.838^a^	0.770^b^	0.829^ab^	0.806^ab^	0.837^a^		0.014	<0.05
Ct.Th, (*μ*m)	200^a^	176^b^	194^a^	192^a^	196^a^		0.003	<0.001
BV (mm^3^)	0.242^a^	0.222^b^	0.239^a^	0.231^ab^	0.241^a^		0.004	<0.05

Data represent the group means. *P*-values are for one-way ANOVA tests. Different letters indicate means that are statistically different based on Tukey-Kramer post hoc test.

**Table 3:** Bone length and mechanical strength of the femur and tibia of mice during ex vivo three-point testing.

Parameters, (units)	Treatments					
	SHM	OVX	S + V	O + G	SVG	*P*-value
Right Femur	(*n* = 11)	(*n* = 12)	(*n* = 8)	(*n* = 14)	(*n* = 9)	
Bone length, (mm)	15.81 ± 0.02^b^	16.26 ± 0.02^a^	15.92 ± 0.02^ab^	16.22 ± 0.03^a^	15.76 ± 0.03^b^	<.0001
Ultimate Force, (N)	16.0 ± 0.3^a^	13.4 ± 0.3^b^	16.1 ± 0.6^a^	15.7 ± 0.5^a^	16.6 ± 0.3^a^	<.0001
Failure Force, (N)	12.1 ± 1.1^a^	5.8 ± 1.0^b^	9.6 ± 1.7^ab^	9.0 ± 1.1^ab^	11.7 ± 1.7^a^	<0.010
Yield Force, (N)	12.3 ± 0.6^a^	10.3 ± 0.4^b^	12.2 ± 0.4^ab^	11.9 ± 0.6^ab^	12.0 ± 2.6^ab^	<0.001
Stiffness, (N/mm)	81.4 ± 1.4	75.8 ± 2.7	80.2 ± 3.1	83.0 ± 2.4	84.4 ± 1.2	NS
Energy to Ultimate Force, (mJ)	3.1 ± 0.1	2.7 ± 0.2	3.0 ± 0.2	3.0 ± 0.2	3.3 ± 0.1	NS

Left Tibia	(*n* = 11)	(*n* = 15)	(*n* = 12)	(*n* = 18)	(*n* = 10)	
Bone length, (mm)	18.08 ± 0.07^bc^	18.38 ± 0.06^a^	18.26 ± 0.03^abc^	18.33 ± 0.06^ab^	18.07 ± 0.04^c^	<0.001
Ultimate Force, (N)	10.5 ± 0.2	9.8 ± 0.2	10.6 ± 0.2	10.4 ± 0.3	10.7 ± 0.4	NS
Failure Force, (N)	4.9 ± 0.9	3.7 ± 0.6	3.3 ± 0.3	4.5 ± 0.6	3.7 ± 0.8	NS
Yield Force, (N)	10.0 ± 0.2	9.1 ± 0.3	10.3 ± 02	9.9 ± 0.3	10.2 ± 0.4	NS
Stiffness, (N/mm)	36.2 ±1.1	35.8 ± 0.8	39.1 ±1.1	37.4 ± 0.8	38.5 ± 0.9	NS
Energy to Ultimate Force, (mJ)	2.0 ± 0.1	1.8 ± 0.1	1.8 ± 0.1	1.9 ± 0.1	1.9 ± 0.1	NS

Data represent the group means ± SEM. Different letters indicate means that are statistically different. NS indicates not significant. Samples that rolled on the supports during mechanical testing were eliminated from the analyses.
